# Bioactive Compounds in Mandarin (*Citrus reticulata*): Composition, Analytical Characterization and Therapeutic Potential

**DOI:** 10.3390/antiox15101220

**Published:** 2026-09-22

**Authors:** Esra Gölcü, Keziban Yazici

**Affiliations:** Department of Horticulture, Faculty of Agriculture, Recep Tayyip Erdoğan University, 53300 Rize, Türkiye; keziban.yazici@erdogan.edu.tr

**Keywords:** mandarin, phytochemicals, metabolomics, nutraceuticals

## Abstract

Mandarin is one of the most widely consumed citrus fruits worldwide and is a rich source of phytochemical compounds with important biological activities for human health. This review comprehensively addresses the distribution, chemical properties, and therapeutic significance of the main bioactive compounds found in mandarins, particularly flavonoids, phenolic acids, carotenoids, vitamins, and essential oils. Furthermore, it presents up-to-date information on the chromatographic, spectroscopic, and metabolomic analytical approaches used to identify and quantify these compounds. The biological activities of mandarin-derived phytochemicals have been evaluated in terms of their antioxidant, anti-inflammatory, cardiovascular, anticancer, and metabolic regulatory effects. Current studies indicate that hesperidin, narirutin, nobiletin, tangeretin, and various phenolic acids make significant contributions to the positive health effects of mandarins. Furthermore, the distribution of these bioactive compounds in different fruit tissues, their concentration levels, and their interactions have been explained, and current scientific evidence regarding their potential effects on human health has been evaluated. In conclusion, this review brings together the latest information on the chemical characterization, therapeutic potential, and analytical methods of bioactive compounds found in mandarins, providing a scientific basis for future research and the development of functional foods, nutraceuticals, and natural health products.

## 1. Introduction

The mandarin (*Citrus reticulata*) is an ancient citrus fruit known for its richness in nutrients and bioactive compounds, as well as its potential medicinal value [1]. With various varieties and hybrids belonging to the Rutaceae family, it is currently one of the most economically important crops in terms of area and production value worldwide [2]. The high nutritional value and functional components of mandarins have led to an increase in research into this fruit in recent years, both in the fields of food science and health sciences. Like other citrus species, mandarins are native to the subtropical and tropical regions of Asia, particularly China and Cochinchina [3,4]. Some researchers have reported that mandarins evolved in a region encompassing Vietnam, South China, India and Japan, alongside other citrus species [5,6]. Thanks to its historical origins and wide adaptability, mandarin is now grown in many parts of the world and plays a significant role in the citrus trade.

Recently, whilst there has been a decline in the consumption and global marketing of citrus fruits with difficult-to-peel skins, such as oranges, grapefruit and pomelos, there has been an increase in the consumption of mandarins, which are easy to peel [7]. Mandarin’s pleasant taste is further enhancing its popularity worldwide [8].

Mandarin production worldwide has seen a significant increase in recent years and accounts for a substantial share of total citrus production. According to FAO data, mandarin production has exceeded 51 million tons, with the area under cultivation reaching millions of hectares. Many countries, notably China, India, Pakistan, Türkiye and Spain, are leading producers of mandarins [9]. Thanks to its favorable ecological conditions, Türkiye holds a key position in global mandarin production, with production concentrated particularly in the Mediterranean and Aegean regions, and it is also among the leading countries in terms of exports. This situation demonstrates that mandarin is a strategic product from both an economic and agricultural perspective.

In addition to growing consumer interest, the morphological and nutritional properties of mandarins are also among the key factors contributing to the fruit’s growing importance. The mandarin orange tree is a thorny, evergreen plant that grows to a height of 2–8 m; most varieties reach an average height of 7.5 m. The tree has a dense crown, and its slender branches bear dark green, lance-shaped leaves with a distinct central vein. The leaf stalks are narrowly winged or slightly marginated. It produces fragrant white flowers, followed by oval or flattened, sweet, juicy, golden-yellow fruits [10]. The mandarin fruit resembles other oranges but is smaller and flatter, with orange-colored, thin, loose, easily peelable skin. The fruit can be up to 8 cm in diameter, with segments that separate easily [11].

Mandarins are a rich source of vitamins C and A, protein, dietary fiber, and essential minerals such as calcium, potassium, phosphorus, and magnesium. They also contain small amounts of B vitamins (B1, B2, B3, B5, B6, B9) and vitamin E. On average, 100 g of mandarin oranges consist of 85% water (85.2 g), 13% carbohydrates (13.34 g), 0.81 g protein, 0.38 g dietary fiber, and 0.31 g fat [5,10].

Mandarins are highly appreciated and preferred by consumers due to their pleasant taste and high content of phytochemicals. As more people become increasingly health-conscious, demand for citrus fruits such as mandarins and their byproducts is steadily increasing [10].

It is important to identify in detail the bioactive compounds found in mandarins and to elucidate their effects on health. The aim of this review is to examine the analytical characterization of phytochemical components such as flavonoids, phenolic acids, carotenoids and essential oils found in mandarins (*Citrus reticulata*), and to evaluate the protective and therapeutic effects of these bioactive compounds on human health in the light of the current scientific literature. This study is not a systematic review but aims to provide a comprehensive synthesis of the research found in the available literature. Unlike previous review articles, which have mainly focused on either phytochemical composition or biological activities, this review integrates phytochemical composition, analytical characterization techniques, and therapeutic properties within a single comprehensive framework. Furthermore, recent advances in metabolomics, chromatographic technologies, and spectroscopic characterization are critically evaluated and compared to provide an up-to-date reference for future functional food and nutraceutical research. In this respect, the study aims to integrate information scattered throughout the literature to create a comprehensive reference source for researchers, highlight current knowledge gaps, and provide directions for future analytical and biomedical studies.

### Literature Search Strategy

This review was conducted as a narrative review with the aim of providing a comprehensive and critical overview of the current scientific literature on the bioactive compounds, analytical characterization and potential health effects of mandarins (*Citrus reticulata*). To enhance transparency in the identification and selection of relevant literature, a structured literature search strategy was applied, and the study identification and screening process was summarized using the PRISMA 2020 flowchart [12] (Figure 1) The literature search was conducted using the Scopus and Google Scholar databases, covering publications from January 2019 to May 2026, with the final search carried out in May 2026. In the search strategy, the keywords ‘mandarin’ and ‘*Citrus reticulata*’ were used in conjunction with the terms ‘bioactive compounds’, ‘phytochemicals’, ‘analytical characterization’, ‘chromatographic analysis’, metabolomics’, ‘health benefits’ and ‘therapeutic potential’. These keywords were searched separately and in various combinations using the Boolean operators AND and OR. The inclusion criteria for the study are follows: (i) having been published between 2019 and 2026, (ii) being an original research or review article published in English, (iii) availability of the full text and (iv) direct relevance to bioactive compounds, analytical characterization, phytochemical profile or potential health effects of mandarin. Exclusion criteria were defined as publications without a full text available, conference abstracts, letters to the editor, book chapters, theses, duplicate entries, studies published in languages other than English, and research not directly relevant to the scope of the review. The identified studies were evaluated at the level of title, abstract and full text; publications deemed scientifically reliable and directly relevant to the aim of the review were included in the analysis. The selected studies were examined from a critical perspective and evaluated comparatively, particularly with regard to the phytochemical composition of mandarins, analytical characterization methods, biological activities and health effects.

## 2. Key Bioactive Compounds in Mandarins

The mandarin fruit is a rich source of phytochemicals, containing both primary and secondary metabolites [13] (Figure 2). The primary metabolites of this fruit consist of macronutrients such as sugars, organic acids, lipids and vitamins, whilst the secondary metabolites comprise phenolic compounds and flavonoids that determine the fruit’s flavor profile and health benefits [14]. These components will be discussed in detail in the following subsection.

### 2.1. Primary Metabolites

The primary metabolites or macronutrients of the mandarin fruit namely carbohydrates (sugars), organic acids, lipids, fatty acids, proteins and amino acids; have a decisive influence on the fruit’s flavor profile, ripeness level and organoleptic qualities (Table 1).

#### 2.1.1. Carbohydrates (Sugars)

One of the key indicators of fruit quality is the balance between sucrose, glucose and fructose, which constitute the carbohydrate structure. Previous studies [15] have confirmed that these sugar ratios are significantly influenced by genetic factors as well as climatic and environmental factors (terroir effect).

The flavor of mandarin oranges is primarily determined by the balance of sugar and acid in the fruit segments. This value, defined as the total soluble solids (TSS)/titratable acidity (TA) ratio [16], is a critical parameter determining the sensory quality of all citrus species [17]. In determining the optimal harvest period, titratable acidity data, in particular, is a decisive factor [18]. The fruit’s ripening process is a dynamic transformation characterized by an increase in TSS values and a decrease in acidity; this change leads to variations in fruit flavor depending on its stage of development. Due to this mechanism, mandarins in the early season have a more acidic profile, while late-season fruits have a sweeter profile. TSS levels, which are approximately 90 g/L at the start of the harvest, can reach up to 150 g/L by the end of the process [7].

Analysis of the total soluble solids (TSS) content of mandarin fruit reveals that approximately 80% of this value consists of sugars, 10% of organic acids, and the remaining 10% of nitrogenous compounds (amino acids). The carbohydrate profile consists primarily of reducing sugars such as glucose, fructose and sucrose [16,19].

#### 2.1.2. Organic Acids

Organic acids are key primary metabolites that, together with sugars, contribute to the characteristic flavor of mandarins. In the organic acid fraction, the dominant component is citric acid, which accounts for 85–90% of the total acid composition; this is followed by malic, succinic and oxalic acids [16,19]. Organic acids not only determine sensory characteristics but also influence fruit quality and the ripening process. When assessed alongside total soluble solids (TSS), the acidity level in the fruit is recognized as a key quality criterion determining the fruit’s ripeness index and consumer preferences. In particular, a high citric acid content can negatively affect consumer acceptance and market value in mandarins. Consequently, various methods have been developed to reduce citric acid levels; however, concerns have been raised regarding the reliability and safety of some of these methods [20].

Compared to orange varieties, mandarins generally exhibit a sweeter and lower-acidity sensory profile due to their higher TSS and lower TA values. In mandarin varieties with the highest consumer acceptance, TSS and TA values of typically 130 g/L and 11 g/L, respectively, are observed, corresponding to a TSS/TA ratio of approximately 13. Deviations from this ratio negatively affect flavor balance; for example, a low TSS/TA ratio of 6.4 leads to an excessively acidic (sour) taste, whilst very high ratios reduce the fruit’s characteristic flavor intensity, resulting in a blander taste profile [16]. In addition to agricultural practices, environmental factors have a significant impact on sugar levels in mandarin fruit. Low temperature and humidity increase sugar concentration, whilst high light intensity has a reducing effect. The ripeness index, expressed as the TSS/TA ratio, also varies depending on climatic conditions; it ranges from 17.5 in a subtropical humid climate to 22.9 in a semi-arid climate. In arid conditions, a higher maturity index stems from the accumulation of soluble substances due to osmotic modification linked to water deficiency stress [21], whereas a lower index is observed in humid climates [22]. This is associated with lower organic acid utilization under humid conditions and consequently, higher acidity [14].

#### 2.1.3. Lipids

Mandarin orange seeds possess significant economic value in terms of the utilization of waste by-products, owing to their rich lipid and oil content [23]. The lipids in mandarin seeds consist of fatty acids, waxes, isoprenoid hydrocarbons, acylglycerols and carotenoids/sterols [24]. These lipids consist of 96.3% neutral fractions (primarily triacylglycerols, 70.5%) and 3.7% polar fractions [25]. Mandarin has a high lipid content of 0.31 g/100 g compared to other citrus species [26]. In general, citrus seeds contain approximately 34–42% oil and are recognized as an important source of essential fatty acids and tocopherols, with potential applications for both human and industrial use [27,28]. Citrus seed oil is a mixture of glycerides containing various fatty acids, the main forms of which are palmitic, stearic, oleic, linoleic and linolenic acids [28].

Furthermore, palmitic, oleic, linoleic and linolenic acids are the principal fatty acids in citrus pulp lipids and citrus seed oil. In mandarin seeds, linoleic acid (42.3%) is the predominant fatty acid, whilst palmitic acid (22–30%) is another important component [23,28,29].

In mandarin pulp, total lipids and phospholipids increased during the early stages of ripening and subsequently decreased; glycolipids, however, increased during ripening and storage [30]. The levels of phospholipids and phytosterols in mandarin seeds are slightly lower than those in oranges but higher than those in grapefruit. In terms of phytosterol levels, mandarin exhibits a biochemical profile comparable to that of oranges and grapefruit [29].

#### 2.1.4. Protein

Compared with orange (6.39–7.09%) and lemon (7.06–11.53%) varieties, mandarin peel has the lowest protein content among citrus fruits, ranging from 2.3% to 3.2% [31]. The fact that the fruit’s protein percentage remains at very low levels of 0.08% implies that mandarin is far from being a significant source of protein [5]. It has been reported that approximately 56 proteins identified in mandarins are predominantly located in the cytoplasm and regulate cellular processes associated with fruit quality [32]. The free amino acid profile in citrus fruits (alanine, arginine, asparagine, aspartic acid, glutamic acid, glycine, serine and proline) consists largely of non-essential amino acids; in particular, proline stands out as the predominant amino acid in fruit juices. However, it is generally accepted that the contribution of these free amino acids to human nutrition is limited [5].

#### 2.1.5. Vitamins

Vitamins are organic micronutrients essential for organisms to maintain normal physiological functions [33]. As vitamins generally cannot be synthesized by organisms, they must be obtained through the diet; this makes citrus fruits an important source of vitamins. Among the fat-soluble and water-soluble vitamins found in citrus fruits, the most representative components are vitamin E and vitamin C, respectively. Furthermore, vitamin A is present in citrus fruits in the form of provitamin A carotenoids, with carotene and β-cryptoxanthin standing out as the primary precursors [5]. Vitamin C is recognized as one of the most important water-soluble antioxidants in citrus fruits and may exhibit protective effect against various diseases, particularly cancers of the digestive system (e.g., stomach and bowel cancers). It also plays a role in protecting intracellular and extracellular components by inhibiting the formation of free radicals and lipid peroxides [34] (Figure 4).

Citrus juices are important beverages widely consumed worldwide due to their high vitamin C content [35]. The vitamin content of citrus fruits is influenced by numerous factors, including variety, ripeness, growing and storage conditions, and rootstock. Reference [36] investigated the effect of citrus rootstocks on the vitamin content and antioxidant activity of the fruit and reported that the observed differences were largely attributable to the rootstock, whilst the level of ripeness had a more limited effect. In terms of vitamin composition, mandarin pulp is a significant source of vitamins A (3%) and C (13%) [13,37]. Vitamin C accounts for approximately 80.5% of the mandarin fruit’s total antioxidant capacity [10] and plays a fundamental role in the formation of connective tissues and the biosynthesis of neurotransmitters [38]. When comparing citrus juices in terms of vitamin C content, orange (535 mg/L) ranks first, followed by grapefruit (384 mg/L), mandarin (372 mg/L) and lemon (337 mg/L) [16].

### 2.2. Secondary Metabolites

In addition to being rich in primary metabolites, mandarin fruit contains a wide range of secondary metabolites, such as polyphenols (particularly flavonoids and phenolic acids), carotenoids and essential oils (Table 1). These components account for the fruit’s distinctive flavor and, as with other citrus fruits, its bioactive properties and potential health benefits [27,39].

Polyphenols are classified into flavonoids and non-flavonoid compounds (phenolic acids) based on the number of hydroxyl groups attached to their aromatic rings [12]. These compounds play a role in various physiological processes in plants, such as growth, development and defense mechanisms, thereby exhibiting diverse biological effects. Furthermore, polyphenols play a significant role in the formation of food quality and flavor characteristics, and, thanks to their antioxidant activities, offer potential contributions to the prevention of chronic diseases [40].

#### 2.2.1. Flavonoids

Natural plant polyphenols are secondary metabolites that play a critical role in plants’ defense mechanisms and are found in a wide variety of citrus fruits; within this group, flavonoids constitute the most important bioactive components [41,42,43]. Flavonoids, which are low-molecular-weight phenolic compounds primarily possessing a (C6-C3-C6) structure, are classified structurally into flavanones, flavones, flavonols and anthocyanins; whilst neohesperidoside derivatives (such as naringin and neohesperidin) impart the characteristic bitter taste of citrus fruits, the rutinoside forms (such as hesperidin and narirutin) are generally tasteless. These bioactive compounds, with their significant nutritional health benefits in terms of therapeutic and nutraceutical potential, help reduce the risk of many chronic diseases and may exhibit anti-inflammatory, antihypertensive and anti-atherosclerotic effects [44,45]. Flavonoids are also important due to their antioxidant, anti-inflammatory, anti-mutagenic and anti-carcinogenic effects [46] (Figure 4). The flavonoid content in citrus juices varies; mandarin juice (~360 mg/L) is slightly higher than that of lemon but lower than that of orange and grapefruit [16]. The flavonoid content varies significantly among mandarin varieties, with hesperidin being the predominant component (53–75%). Hesperidin is associated with anti-inflammatory, antihypertensive and anticancer effects [13,47]. In particular, hesperidin stands out as the most abundant flavonoid in species such as mandarin.

Polymethoxyflavones (PMFs) are compounds containing at least two methoxy groups in their chemical structure and are present in their free aglycone form. As they are almost exclusively found in the citrus family, they are unique compounds that can be used as biomarkers for citrus classification. The highest levels are found in mandarin peel, followed by sweet orange peel, whilst lower levels have been detected in lemon and pomelo [48]. PMFs possess important biological activities such as anti-allergic, antioxidant, antibacterial, anti-proliferative and anti-inflammatory properties. It has been reported that mandarin peel is rich in PMFs, with nobiletin (3.1–11.1 mg/g DW) being the predominant compound, followed by tangeretin (1.5–2.3 mg/g DW) and sinensetin (0.1–2.6 mg/g DW) [49]. Thanks to their significant biological activities, such as anti-inflammatory, anti-cancer, antioxidant, anti-lipogenic and metal chelating properties, mandarin flavonoids are positioned as promising bioactive compounds not only in the pharmacological field but also in commercial sectors such as the food, cosmetics and coloring industries [50].

**Figure 2:**
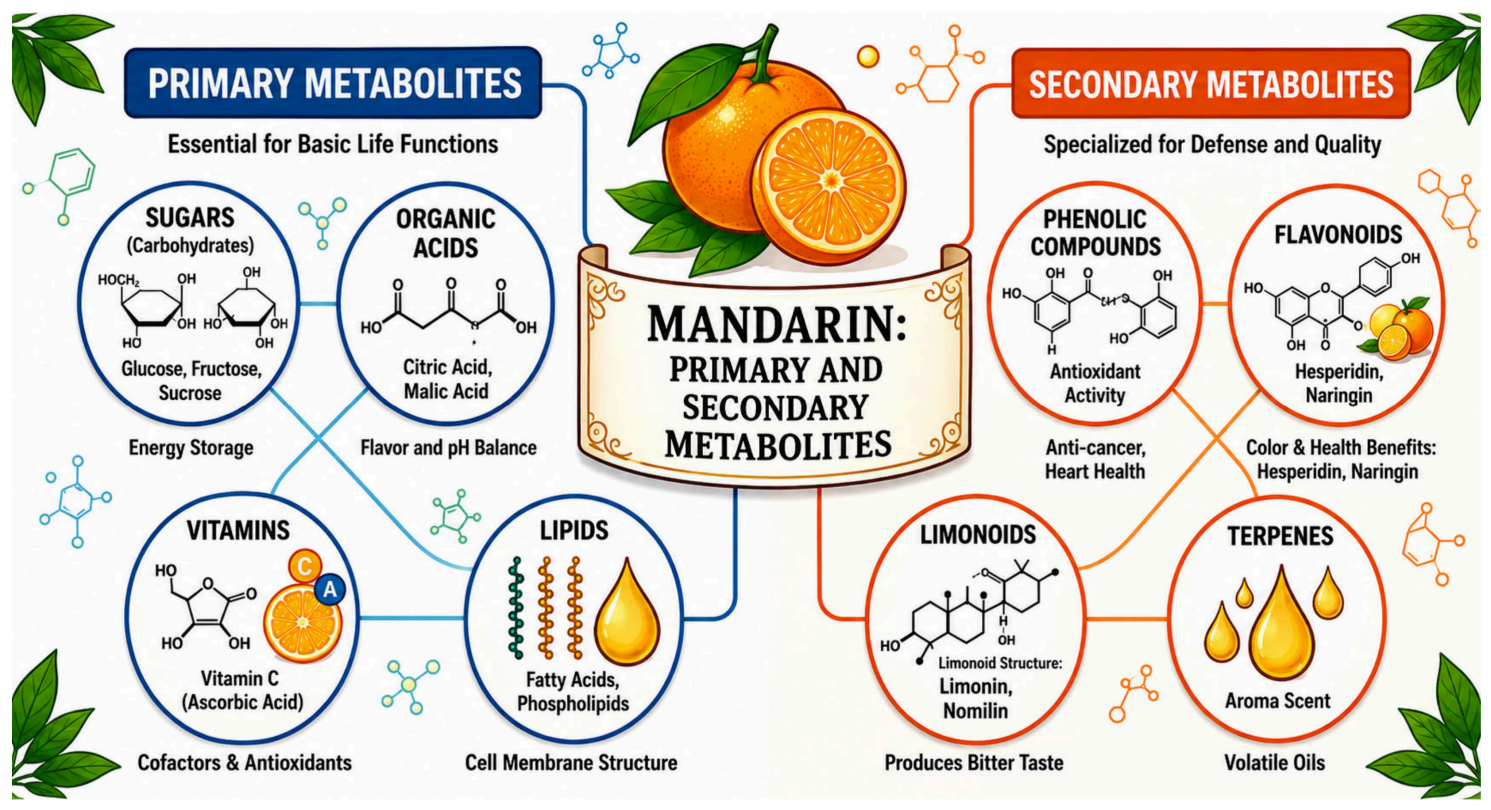
Primary and secondary metabolites in mandarins [10,13,16,31,38,46].

#### 2.2.2. Phenolic Acids

In general terms, phenolic acids are polyphenols comprising two groups: hydroxybenzoic acids (procatechuic, p-hydroxybenzoic and vanillic) and hydroxycinnamic acids (caffeic, p-coumaric, ferulic and sinapic) [51]. The health-protective and health-promoting effects of phenolic acids are well established. In particular, their significantly superior antioxidant capacity compared to known antioxidant vitamins under in vitro conditions is directly related to their biological activities, such as antibacterial, antiviral, anticarcinogenic, anti-inflammatory and antimutagenic effects [51,52] (Figure 4). These multifaceted pharmacological functions clearly highlight the strategic importance of citrus-derived phenolic acids in the pharmaceutical industry.

The phenolic acid profile and concentration in citrus species vary significantly depending on factors such as genetic variation, the anatomical parts of the fruit, and stages of physiological ripeness. Studies have shown that the peel (pericarp) of semi-ripe fruits contains higher levels of phenolic acids compared to the fruit flesh. [53]. When evaluated in terms of chemical classification, hydroxycinnamic acids (ferulic, p-coumaric, caffeic, sinapic and chlorogenic acids) have been identified as the predominant forms in various anatomical parts of the fruit. In contrast, hydroxybenzoic acid derivatives such as vanillic, p-hydroxybenzoic and gallic acids were found to be present in lower amounts in the pericarp tissue [54].

The peel of the mandarin fruit exhibits a profile particularly rich in hydroxycinnamic acids; here, ferulic acid (67%) is the dominant component, whilst p-coumaric (9%), caffeic (5.5%) and sinapic (5.5%) acids are present in lower proportions. In addition, it has been reported that hydroxybenzoic acids are present in lower proportions in the peel tissue; specifically, vanillic acid (5.5%) and protocatechuic and p-hydroxybenzoic acids (less than 3%) [10]. A similar composition distribution has also been observed in mandarin juices from different genotypes (Satsuma, Robinson and Fremont). Among the hydroxycinnamic acids in these fruit juices, ferulic acid (5.4–20.8 mg/L FW) was again found to be present in the highest concentration; followed by chlorogenic acid (0.88–5.0 mg/L FW), caffeic acid (0.71–2.7 mg/L FW) and p-coumaric acid (0.17–2.3 mg/L FW) [55].

#### 2.2.3. Carotenoids

Carotenoids are the primary factor determining the color of various citrus fruits [56]. In citrus species, approximately 115 different carotenoids have been identified, including carotenes such as α-carotene, β-carotene and lycopene, as well as xanthophylls such as β-cryptoxanthin, lutein, zeaxanthin and violaxanthin [57]. Carotenoid content and composition vary depending on the citrus variety, ripeness level, and growing and storage conditions [58]. During the ripening process, carotenoid accumulation increases alongside the transformation of chloroplasts into chromoplasts and the breakdown of chlorophyll. Among different citrus species, mandarin is particularly rich in β-cryptoxanthin and violaxanthin and is one of the species with the highest carotenoid content [16,59]. Carotenoid levels can reach 25–300 μg/g FW in the peel and 10–40 μg/g FW in the fruit flesh. Generally, luteoxanthin predominates in the mandarin peel, whilst antheraxanthin and zeaxanthin are prominent in the fruit flesh [13,16]. In addition to their role in color formation, carotenoids have become an important subject of research due to their protective effects against diseases such as cancer, cardiovascular diseases and diabetes, owing to their role as precursors of vitamin A and their potent antioxidant properties [60] (Figure 4).

#### 2.2.4. Essential Oils

The release of volatile compounds increases with rising temperature, the degree of ripeness, and damage to the peel and pulp tissues [30]. Over 300 volatile compounds and oils have been identified in citrus fruits; these consist primarily of terpenoid hydrocarbons (monoterpenes and sesquiterpenes), esters, aldehydes, ketones, alcohols and volatile organic acids [61]. Citrus essential oils are one of the most popular groups among natural essential oils and are of high commercial importance. In mandarins, essential oils are primarily concentrated in the peel tissue, accounting for approximately 1% of the fruit’s weight. These volatile compounds form the basis of the aroma profile of both the peel oil obtained by cold pressing and the fractions of the fruit juice, and they determine the characteristic properties of the mandarin [62].

Mandarin peels contain primarily monoterpene hydrocarbons, which account for 86.62% (*w*/*w*) (weight-weight) of the total essential oil content. However, this proportion is relatively lower than the values reported for other citrus species such as sweet orange, bitter orange and grapefruit. In the essential oil profile of the mandarin, limonene (95%) stands out as the most common volatile compound; this is followed by γ-terpinene (16.4–22.7%), α-pinene (2.0–2.7%), β-pinene (1.4–2.1%), β-myrcene (1.5–1.8%) and linalool (0.67%) [63]. Additionally, other volatile compounds such as α-phellandrene, linalyl acetate, nonanal, trans-limonene oxide, α-farnesene, octyl aldehyde, decyl aldehyde, β-caryophyllene and α-bergamotene were detected in trace amounts (0.06%) [64]. The content of any component of the essential oil in mandarins may vary depending on the variety, stage of ripeness, extraction method used, and the part of the plant utilized [65]. Essential oils are used in various commercial products due to their antimicrobial and insect-repellent properties [6] (Figure 4).

### 2.3. Minerals

Mandarin peel is an important source of nutrition, containing a higher mineral content than the pulp. The peel is rich in macrominerals such as potassium (141 mg/100 g FW), calcium (37.1 mg/100 g FW), phosphorus (19.9 mg/100 g FW) and magnesium (12.9 mg/100 g FW), and these components can provide approximately 10–36% of the daily reference intake. Among these minerals, potassium plays a role in regulating water, electrolyte and acid-base balance; calcium in strengthening the skeletal system; and phosphorus, together with calcium, in the formation of bone and tooth structure [66]. Furthermore, the sodium content of mandarin peel (1.09 mg/100 g FW) is lower than that of the fruit pulp (2.7–6.9 mg/L FW) [10,65].

**Table 1:** The main bioactive compounds found in mandarins, the tissues in which they are found, and their concentration ranges.

Compound Group	Representative Compound	Tissue Location	Concentration Range	Reference
Carbohydrates	Sucrose	Pulp	4.25–8.64 g/100 g FW	[13,67]
	Glucose	Pulp	1.81–3.00 g/100 g FW	[13,67]
	Fructose	Pulp	1.91–3.53 g/100 g FW	[13,67]
Protein	-	Pulp	0.50–1.00 g/100 g FW	[31,67]
Lipids	-	Pulp Seed	0.17–0.42 g/100 g FW 0.31 g/100 g FW	[26,67]
Minerals	Calcium	Peel	18.00–48.00 mg/100 g FW	[66,67]
	Phosphorus	Peel	17.00–22.00 mg/100 g FW	[66,67]
	Potassium	Peel	130.00–196.00 mg/100 g FW	[66,67]
Vitamins	Vitamin C	Pulp	22.10–34.00 mg/100 g FW	[10,67]
	Vitamin A	Pulp	34.00 µg/100 g FW	[37,67]
Flavonoids	Hesperidin	Peel	660.26–924.05 mg/100 g FW	[68,69]
	Narirutin	Peel	9.60–28.81 mg/100 g FW	
	Nobiletin	Peel	115.95–158.08 mg/100 g FW	
	Tangeretin	Peel	32.85–46.99 mg/100 g FW	
Essential oils	Limonene	Peel	65.00–94.00%	[70,71]
	Linalool	Peel	1.65–2.31%	
	γ–Terpinene	Peel	1.79–17.30%	
Carotenoids	β–Carotene	Peel and pulp	0.03–0.45 µg/100 g FW	[13,67]
	α–Carotene	Peel and pulp	0.01–0.46 µg/100 g FW	[13,67]
	β–Cryptoxanthin	Peel and pulp	0.04–0.49 µg/100 g FW	[13,67]

## 3. Instrumental Analytical Methods and Characterization

Like other fruits, mandarins consist of a wide variety of chemical compounds. Therefore, it is of great importance to develop analytical methods that maintain high selectivity in order to determine different bioactive compounds with high accuracy and sensitivity, even at low concentration levels. Various analytical approaches are available in the literature for this purpose (Figure 3).

Chromatographic methods are currently the most widely used techniques for the determination of bioactive compounds in mandarins. However, research has also been conducted to develop alternatives that are more cost- and time-effective. In this context, the use of spectroscopic techniques, and to a lesser extent titrimetric methods, is particularly noteworthy [72,73]. Among titrimetric methods, the titration method based on 2,6-dichlorophenolindophenol is widely applied for the quantitative determination of ascorbic acid [74]. Although spectrophotometric methods are at a disadvantage in terms of selectivity compared to chromatographic techniques, they are frequently preferred for determining the total amount of certain groups of bioactive compounds due to their low cost and ability to provide rapid results.

### 3.1. Spectrophotometry

Spectrophotometry is a cost-effective screening method widely used in both industry and scientific research, enabling the rapid and practical identification of various compounds (Table 2). Studies in this context focus on determining the total phenolic, total flavonoid and total carotenoid contents found in different parts of the mandarin fruit (peel, juice, seeds and leaves) using spectrophotometric methods.

Among methods other than chromatographic techniques, spectrophotometry is one of the most widely used approaches [75,76]. In recent studies, this technique is used not only for determining total quantities but also for comparing the effectiveness of different extraction methodologies and to characterize the fruit’s bioactive potential.

Spectrophotometric methods play an indispensable role in both industry and scientific research in the characterization of the phytochemical profile and functional food value of mandarin fruit, owing to their advantages such as low cost, speed and ease of application. In this context, the traditional Folin–Ciocalteu assay (total phenolic content) [77] and the aluminum chloride colorimetric method (total flavonoids) [78] continue to serve as the primary reference methods. However, in the determination of antioxidant capacity, a combination of spectrophotometric tests with different mechanisms such as DPPH, ABTS, FRAP [77] and in particular, CUPRAC, which exhibits high matrix stability is gaining prominence in the current literature. Regarding carotenoid determination, alongside classical approaches such as those by Lichtenthaler and Hornero-Méndez, the use of derivative spectroscopy and different solvent systems particularly in the analysis of specific pigments such as β-cryptoxanthin offers a practical alternative to chromatographic techniques (HPLC) by enhancing selectivity [79]. Overall, when supported by chemometric models, these optical methods play a significant role as a comprehensive and cost-effective characterization tool in determining the harvest time of mandarins, verifying geographical indications, and quality control processes.

### 3.2. Chromatographic Techniques

Mandarin samples possess a complex matrix structure containing a large number of bioactive compounds. Therefore, in order to obtain reliable and accurate results, appropriate sample preparation and pre-treatment techniques must be applied prior to analysis. Following these pre-treatments, chromatographic separation techniques are generally employed, which significantly enhances the selectivity of the analysis.

The primary chromatographic methods used for the identification of bioactive compounds include high-performance liquid chromatography (HPLC), ultra-high-performance liquid chromatography (UHPLC/UPLC), thin-layer chromatography (TLC) and gas chromatography coupled with mass spectrometry (GC-MS). These methods have a wide range of applications in the separation and identification of different groups of compounds. HPLC is a technique widely used in the analysis of a wide variety of compounds, such as organic acids, amines, polymethoxylated flavonoids, flavanone glycosides, carotenoids and polyphenols. Thanks to its high resolution and reproducibility, it provides reliable results in complex plant matrices. In contrast, gas chromatography (GC) methods are more suitable for the analysis of volatile and semi-volatile compounds and are particularly preferred for the determination of aroma components specific to mandarin.

#### 3.2.1. High Performance Liquid Chromatography (HPLC)

Various detector systems are used in chromatographic analyses to achieve acceptable levels of sensitivity and selectivity. HPLC is most commonly used in conjunction with a diode array detector (DAD), a UV detector and a mass spectrometer (MS). In particular, MS detectors offer significant advantages due to their ability to provide high sensitivity and selectivity in the analysis of compounds such as carotenoids and polyphenols (Table 2). When a DAD is used, different compound groups can be identified at their characteristic wavelengths; for example, ascorbic acid at 254 nm, organic acids at 214 nm, flavonoids and polymethoxylated flavonoids (PMFs) at 330 nm, hesperidin at 455 nm, amines at 223 nm, and carotenoids at 450 nm. In the HPLC analysis of bioactive compounds, mobile phases consisting of a mixture of C18 stationary phase and acidic solutions of acetonitrile (phosphoric acid, acetic acid or ammonium acetate) are generally used. The pH value is a critical parameter, particularly in the analysis of organic acids, and a sodium dihydrogen phosphate solution adjusted to approximately pH 2.8 is generally preferred [80,81]. Although the analysis of different compounds on the same column is challenging, amines (octopamine, synephrine, tyramine) and organic acids (ascorbic acid, citric acid) can be separated simultaneously using a C18 column and a low-concentration phosphoric acid solution [82]. However, in the analysis of phenethylamine alkaloids, mobile phases consisting of a mixture of acetonitrile and ammonium acetate, combined with the use of a PFP (pentafluoro phenyl) column to prevent the interaction of amines with silanol groups, yield more successful results [83,84].

For the determination of polymethoxylated flavonoids (tangeretin, nobiletin, sinensetin), a C18 column and a formic acid–acetonitrile mixture yields effective results [85]. Carotenoid analysis, however, is more complex due to isomeric diversity; C18 columns may prove inadequate for the isomeric separation of carotenes and xanthophylls due to weak interactions and insufficient surface penetration [86]. As C18 columns are limited in the analysis of carotenoids, triacontyl (C30) columns, which provide high resolution, are preferred. When used with aqueous mixtures of organic solvents such as methanol and methyl tert-butyl ether (MTBE), these columns can separate carotenoid isomers much more effectively [79,87].

HPLC analyses are typically performed in gradient mode to prevent peak overlap and optimize run time. To obtain comprehensive results, techniques such as LC-ESI-MS/MS and UPLC-DAD are combined; flavonoids are analyzed at 280 nm, whilst polymethoxylated flavonoids are analyzed at 330 nm. Today, UPLC stands out as a faster, more efficient and more sensitive method than HPLC for the determination of citrus components [88,89,90] (Table 2). A UPLC-MS/MS method reported in previous study [88] is capable of simultaneously analyzing 66 compounds in satsuma mandarins with high selectivity and sensitivity in just 15 min. This study demonstrates UPLC’s capability for rapid and comprehensive analysis in complex plant matrices.

#### 3.2.2. Gas Chromatography (GC)

Gas chromatography is a rapid and practical analytical method widely used for the identification of volatile aroma compounds found in citrus fruits [91,92]. The separation of aroma compounds by GC is generally carried out on fused silica-based capillary columns coated with polar polyethylene glycol. Quantitative analysis of volatile compounds is mostly performed using mass spectrometry (GC-MS) in single ion monitoring (SIM) mode or with a flame ionization detector (GC-FID) (Table 2).

Studies have demonstrated that GC techniques are effective in determining the volatile profile of mandarins. For example, [93] identified and quantified a total of 47 volatile compounds in the juice of five different mandarin varieties. Gas chromatography–olfactometry (GC-O) is also widely used to identify aroma-active compounds in mandarin samples. As the aromatic quality of the fruit is largely dependent on aroma-active compounds, the GC-O method enables the identification of the compounds that contribute most to the overall aroma. The main GC-O approaches used for this purpose are dilution analysis, detection frequency and perceived intensity methods [94].

When analyzing more complex samples, multidimensional gas chromatography (MDGC) offers significant advantages. This technique is based on the sequential application of two or more chromatographic separations, and the separation process can be carried out in either heart-cut 2-D GC–GC or comprehensive 2-D GC modes. In the split-column approach, specific compounds selected from the first dimension are transferred to the second dimension; in comprehensive 2-D GC, all components present in the sample are directed to the second column via a system known as a ‘modulator’ [95]. In a study by [93], the enantiomeric distribution of chiral compounds in mandarin essential oils was determined by comparing the traditional chiral gas chromatography–flame ionization detector (enantio-GC-FID) method with the heart-cut multidimensional gas chromatography mass spectrometry (MDGC/MS) method. The results demonstrated that the MDGC/MS method provided more reliable results due to its higher resolution and flexibility, whilst one-dimensional methods could yield erroneous results in certain cases.

### 3.3. Spectroscopic Methods

#### 3.3.1. Nuclear Magnetic Resonance (NMR) Spectroscopy

In the structural characterization of bioactive compounds, particularly when dealing with substances isolated for the first time, Nuclear Magnetic Resonance (NMR) spectroscopy is a widely preferred and effective analytical method [96]. This method provides detailed information on the structural properties of molecules, enabling the reliable identification of functional groups and the molecular skeleton (Table 2).

The effectiveness of NMR spectroscopy in the analysis of citrus components has been demonstrated in various studies. For example, in a study conducted by [97], various bioactive compounds isolated from mandarin flowers were identified using NMR. In this context, the structural characterization of important compounds such as hesperidin, rutin, quercetin, nicotiflorin, eriocitrin, narirutin, phenethyl glucoside and unshuoside A was successfully carried out.

Generally speaking, whilst NMR spectroscopy is an indispensable method for identifying new compounds due to its ability to provide highly accurate structural information, it is mostly used as a complementary analytical tool alongside chromatographic and mass spectrometric techniques, owing to its low sensitivity and high cost.

#### 3.3.2. Fourier Transform Infrared Spectroscopy (FTIR)

One of the methods used to a more limited extent in the analysis of bioactive compounds in mandarin fruit is Fourier transform infrared spectroscopy (FTIR). This technique is based on the vibrational movements of molecules and operates on the principle that infrared radiation absorbed by the sample induces vibrations in molecular bonds(Table 2). These vibrations cause changes in the bond lengths and angular values between atoms. The energy released when the molecule returns to its ground state is recorded as a characteristic spectrum.

As a significant proportion of the bioactive compounds found in fruit are carbon-based, these compounds are active in the infrared region and are suitable for FTIR analysis. This method is used, in particular, to identify functional groups (e.g., alken (C–H), aromatic (C–H) and double bond (C=C)) present in mandarin extracts [98,99]. In a study conducted by [99], FTIR analysis of mandarin essential oil revealed the presence of approximately 10 different volatile compounds and various functional groups. Such information is important in guiding further isolation and characterization studies of the relevant compounds.

However, the FTIR method provides limited information regarding the precise structural identification or quantitative determination of compounds. For this reason, this method is primarily used for screening purposes and is generally recommended to be evaluated in conjunction with other analytical techniques.

Although spectroscopic methods such as FTIR offer certain advantages, they are not commonly preferred for the analysis of bioactive compounds. These methods generally require simpler sample preparation processes and can be carried out using less complex equipment compared to chromatographic techniques. This reduces analysis time and lowers costs. However, particularly when used in conjunction with mass spectrometry, chromatographic methods rank among the most widely used and reliable analytical approaches due to their high accuracy and selectivity.

**Figure 3:**
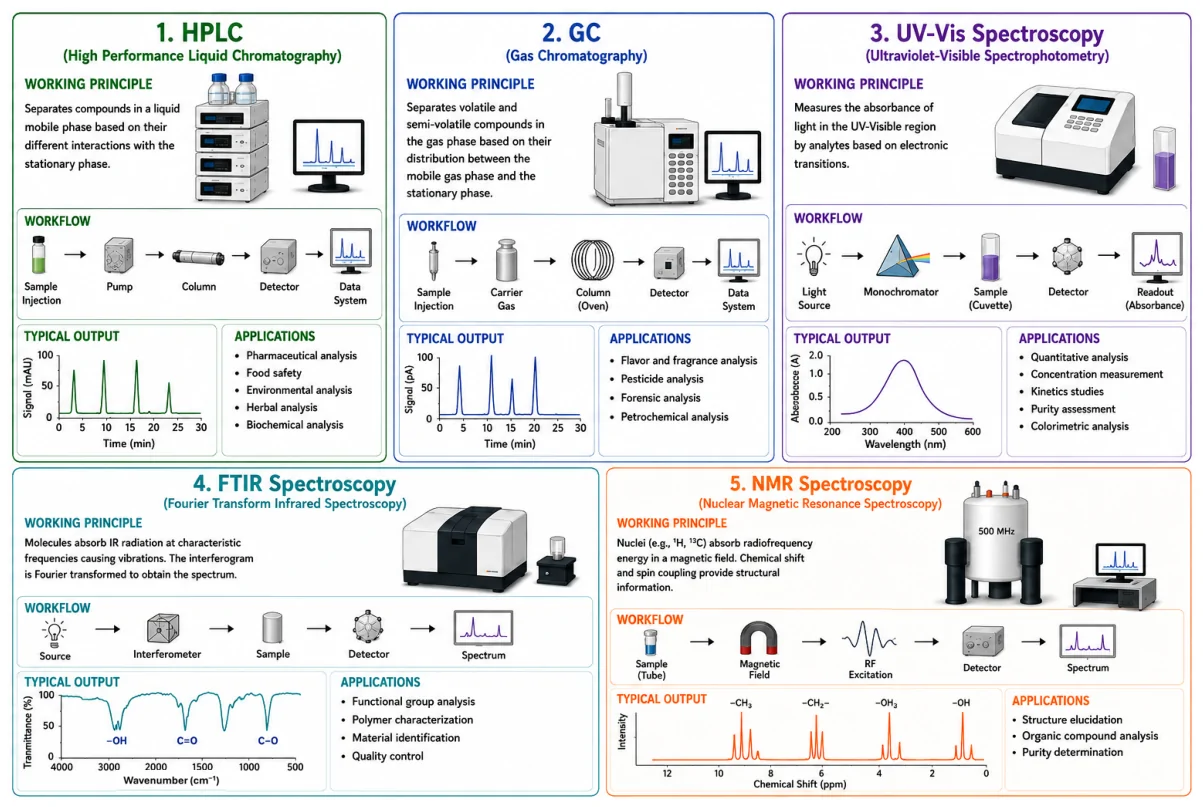
Analytical instruments for the separation, identification and characterization of compounds [75,76,82,84,96,99].

**Table 2:** A comparative evaluation of analytical techniques used in the characterization of mandarin phytochemicals.

Analytical Technique	Target Compound	Main Applications	Advantages	Limitations	Reference
HPLC	Sugars, organic acids, Flavonoids, phenolic acids, carotenoids	Quantitative analysis and quality control	High accuracy, reproducibility	Limited structural validation	[1]
LC-MS/MS	Flavonoids, Polymethoxyflavones, phenolic acids, carotenoids	Structural characterization and precise quantitative determination	High sensitivity and selectivity	High instrumentation and maintenance costs	[100]
UHPLC-QTOF-MS	Complex phytochemical mixtures	Distribution of unknown metabolites	Simultaneous analysis of numerous compounds	Complex data processing	[101]
GC-MS	Aroma compounds, essential oils	Aroma profiling and volatile compound characterization	Excellent separation and identification of volatile compounds	Not suitable for non-volatile compounds	[102]
HS-SPME-GC-MS	Limonene, γ-terpinene, linalool	Aroma analysis	Solvent-free, rapid sample preparation, high sensitivity	Fiber selection sensitive	[103]
FTIR	Pectins, polysaccharides, functional groups	Rapid chemical fingerprinting and quality assessment	Fast, non-destructive, minimal sample preparation	Low specificity	[104]
NMR	Sugars, organic acids, metabolites	Structural characterization and metabolomics	Minimal sample preparation, highly reproducible, comprehensive metabolite profiling	Low sensitivity, high cost	[104]
ICP-MS	Minerals and trace elements	Elemental analysis	Extremely high sensitivity and multi-element detection	High equipment costs	[105]
UV–Vis Spectrophotometry	Total phenolics, flavonoids, antioxidant activity	Rapid screening assays	Simple, inexpensive, high throughput	Limited specificity and susceptibility to matrix interference	[106]

## 4. Therapeutic Effects on Human Health

Previous studies have indicated the potential beneficial effects of different parts of the *C. reticulata* (mandarin) fruit and byproducts derived from this fruit on various diseases and medical conditions [107]. This protective and restorative potential of the mandarin on health is based on the synergistic effect of bioactive molecules such as polyphenolic compounds, vitamins and minerals, which are present in high concentrations. Scientific studies confirm that these components play critical and supportive roles in various physiological mechanisms, including cardiovascular function, metabolic processes, the regulation of inflammation, and oxidative protection at the cellular level (Figure 4).

### 4.1. Effects on Antioxidant and Anti-Inflammatory Properties

Mandarins are not only a delicious and refreshing fruit, but also a highly valuable source of antioxidants for human health. Antioxidants help to prevent or slow down cellular damage by reducing oxidative stress caused by free radicals in the body; in this way, they play an important role in reducing the risk of chronic diseases such as cancer and cardiovascular diseases, as well as in slowing the process. The total antioxidant activity of mandarins stems from the synergistic interaction of the various bioactive compounds they contain; these components are primarily grouped into three main categories: vitamins, carotenoids and phenolic compounds (particularly flavonoids). Both the flesh (pulp) and the peel (flavedo and albedo) of the fruit are rich in these compounds [108]. In particular, vitamin C (ascorbic acid) and carotenoids are the two key components that stand out in the mandarin’s antioxidant profile. Vitamin C, a potent water-soluble antioxidant, neutralizes free radicals, prevents lipid peroxidation and replenishes other antioxidants such as vitamin E, accounting for approximately 20–40 per cent of total antioxidant activity [109]. It also exhibits synergistic interactions with flavonoids and phenolic acids (e.g., sinapic and caffeic acids). Carotenoids found in mandarins, such as β-carotene, lutein, zeaxanthin and β-cryptoxanthin, neutralize free radicals by protecting cell membranes and, together with ascorbic acid, create a powerful antioxidant synergy [110,111]. Furthermore, mandarin peels are extremely rich in flavonoids such as hesperidin, naringin, tangeretin and rutin; these compounds account for approximately 86 per cent of the total phenolics in the peel, offering significant antioxidant potential [112]. There is evidence to suggest that citrus flavonoids such as hesperidin, eriocitrin and narirutin may help reduce the biological burden associated with inflammation by regulating oxidative stress levels [113]. The antioxidant compounds found in citrus fruits play a significant role in cellular protection [114]. Reducing the damage that oxidative stress can cause to DNA, lipid and protein structures may contribute to the preservation of cellular integrity and the slowing down of ageng processes [115].

### 4.2. Effects on the Cardiovascular System

The potential beneficial effects of mandarins on the cardiovascular system are thought to stem from nutrients commonly found in citrus fruits, such as potassium, phenolic compounds, carotenoids and vitamin C [116,117]. Various dietary studies involving citrus consumption have shown that potassium contributes to the regulation of blood pressure and may partially offset the adverse effects of high sodium intake [117,118]. Furthermore, it has been stated that phenolic compounds found in citrus fruits may have positive effects on vascular health and contribute to the preservation of vascular elasticity [116,119]. It has been suggested that flavonoids such as hesperidin may support the preservation of vascular structure by reducing oxidative stress [120]. Furthermore, various studies have shown that vitamin C and carotenoids may reduce the oxidation of LDL cholesterol by regulating oxidative processes, thereby supporting cardiovascular health [121,122]. In addition, there are findings suggesting that the consumption of citrus fruits may lead to improvements in total cholesterol and triglyceride levels in some individuals [123].

### 4.3. Effects on Metabolic Regulation and Obesity

Citrus fruits, which are high in soluble fiber, play a significant role in regulating the postprandial glycemic response [124]. The ability of fiber to delay gastric emptying and slow down the absorption of carbohydrates contributes to preventing sudden fluctuations in blood glucose levels [125,126]. These effects may support the maintenance of glycemic balance, particularly in individuals with insulin resistance. Various studies have demonstrated that flavonoids such as hesperidin and naringin, which are commonly found in citrus fruits, may have positive effects on lipid metabolism and insulin sensitivity [124,127]. Studies have indicated that these compounds can limit fat accumulation in adipose tissue and regulate certain metabolic pathways associated with insulin response [128]. Citrus phytochemicals are considered promising components for the prevention and management of obesity-related disorders. Citrus fruits rank among the primary bioactive sources, particularly notable for their high flavonoid and p-synephrine content. Studies have demonstrated that citrus fruits can exhibit anti-obesity effects through mechanisms such as the regulation of energy intake and expenditure, the modulation of lipid metabolism, and the control of adipogenesis [129]. Furthermore, it has been reported that citrus phytochemicals may reduce the risk of developing obesity [130].

### 4.4. Effects on Cancer

Citrus fruits play a central role in cancer biology research due to their antioxidant, anti-inflammatory and antimicrobial properties. The rich phytochemical composition of citrus species and their high flavonoid concentrations indicate that these fruits exhibit potent anti-cancer activity against various cancer cell lines in preclinical studies. In particular, flavonoids and specific compounds such as nobiletin, found in high concentrations in the peel tissue, have been reported in studies to possess significant anti-tumor activity that inhibits tumor growth in the colon, skin, lung, prostate and liver [131]. Preclinical research on mandarin and mandarin juice extracts clearly elucidates the tumor-suppressing mechanisms of this fruit at the cellular level. It has been observed that mandarin juice extract halts proliferation in leukemia and breast cancer cells, inhibits the cell cycle at the G2/M phase, and induces autophagic cell death. Furthermore, it has been reported that it reduces the migration capacity of tumor cells by modulating matrix metalloproteinase-2 (MMP-2) activity [132]. Furthermore, experimental data have confirmed that dietary protocols enriched with mandarin-derived flavonoids such as hesperidin and β-cryptoxanthin suppress colon cancer and lung tumors in animal models; these protocols also reduce the levels of key inflammatory markers such as TNF-α, IL-1β, IL-6, COX-2 and iNOS [133].

**Figure 4:**
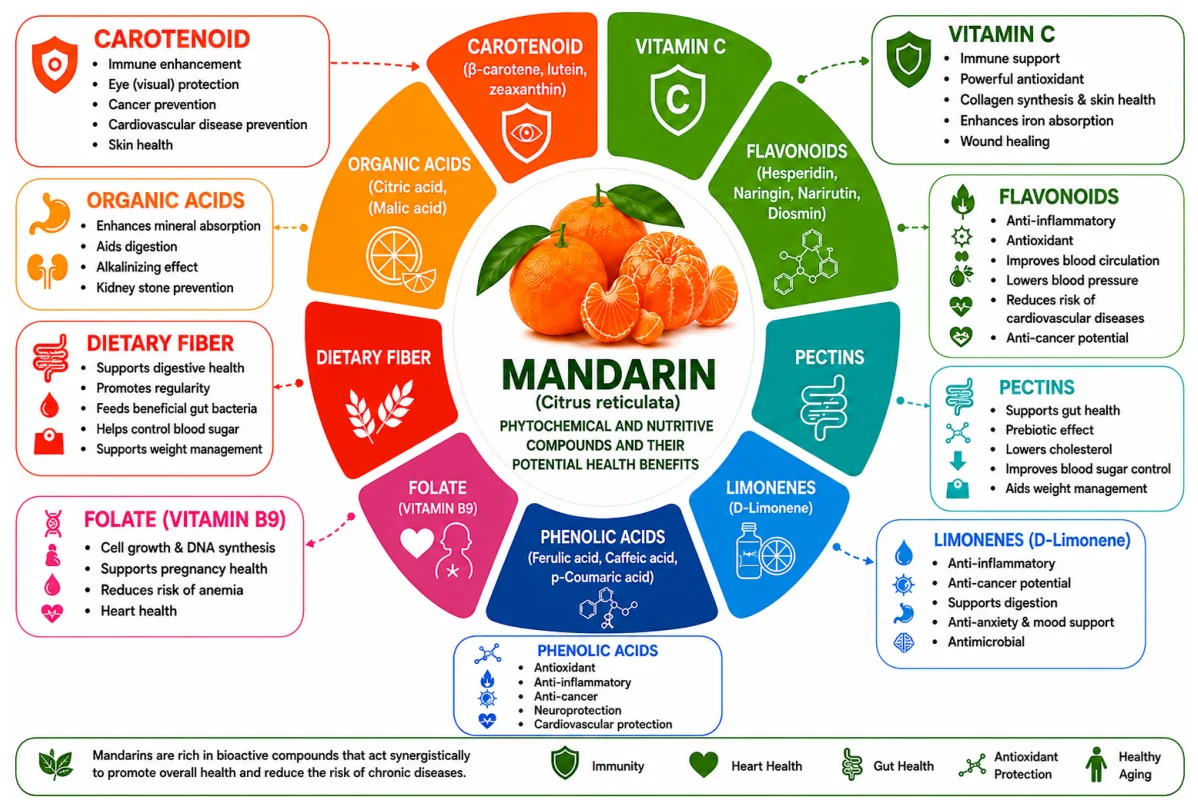
Mandarin phytochemical and nutritive compounds and their potential health benefits [109,110,111,113,116,117,124,131].

## 5. Future Prospects and Conclusions

With growing awareness of healthy eating, the consumption of fruits rich in bio-active compounds has gained significant momentum on a global scale. In this context, the mandarin (*Citrus reticulata*) stands out as a critical functional food component in nutritional science, thanks to its rich content of flavonoids, phenolic acids, vitamins, carotenoids, pectin and essential fatty acids. This review provides a comprehensive and up-to-date synthesis of current knowledge regarding the chemical composition, nutritional value, therapeutic effects and analytical methods of mandarin.

The literature reviewed indicates that the biological activities of mandarins are largely associated with their rich phytochemical profile, particularly flavonoids such as hesperidin, narirutin, nobiletin and tangeretin, which exhibit antioxidant, anti-inflammatory, antimicrobial, anti-diabetic, cardioprotective and anti-cancer properties. Furthermore, the combined presence of carotenoids, phenolic acids, vitamins and other bioactive components enhances the functional value of mandarins and supports their use in the development of functional foods, nutraceuticals, medicines and natural food additives. Another important finding of this review is the evaluation of modern analytical techniques used for phytochemical characterization. Advanced chromatographic and spectroscopic approaches, including HPLC, UHPLC, LC-MS/MS, GC-MS, FT-IR and NMR, enable comprehensive metabolomic profiling whilst ensuring the reliable identification and quantification of individual compounds. The integration of these techniques not only improves analytical accuracy but also strengthens quality control, authenticity assessment and standardization of mandarin-derived products.

Although recent research has significantly expanded our understanding of bioactive compounds in mandarins, further research is needed to fill the existing gaps in our knowledge. Future studies should prioritize the comprehensive characterization of bioactive compounds in different mandarin varieties and fruit tissues and elucidate the molecular mechanisms underlying their absorption, bioavailability, metabolic transformation, stability and biological activity. Furthermore, translating promising findings from experimental models into clinical practice will require robust in vivo studies and well-designed human clinical trials to validate their efficacy and safety. Furthermore, the integration of advanced chromatographic and spectroscopic techniques with multi-omics approaches will enable a more comprehensive understanding of the phytochemical composition of mandarin and accelerate the discovery and application of bioactive compounds in functional foods, nutraceuticals and pharmaceutical products.

In conclusion, mandarin (*Citrus reticulata*) stands out as a valuable functional food owing to its rich phytochemical composition, scientifically established health benefits and broad potential for industrial applications. This review comprehensively presents the current body of knowledge while providing an integrated perspective on phytochemical composition, advanced analytical characterization, biological activities, and therapeutic potential. It also highlights existing research gaps and identifies priorities for future research. The continuation of interdisciplinary research bringing together food science, analytical chemistry, nutrition, biotechnology and clinical sciences will make significant contributions to a better understanding of the mechanisms of action of bioactive compounds derived from mandarins, the scientific validation of their health benefits, and their more effective utilization in the development of functional foods, nutraceuticals and pharmaceutical products.

## Figures and Tables

**Figure 1:**
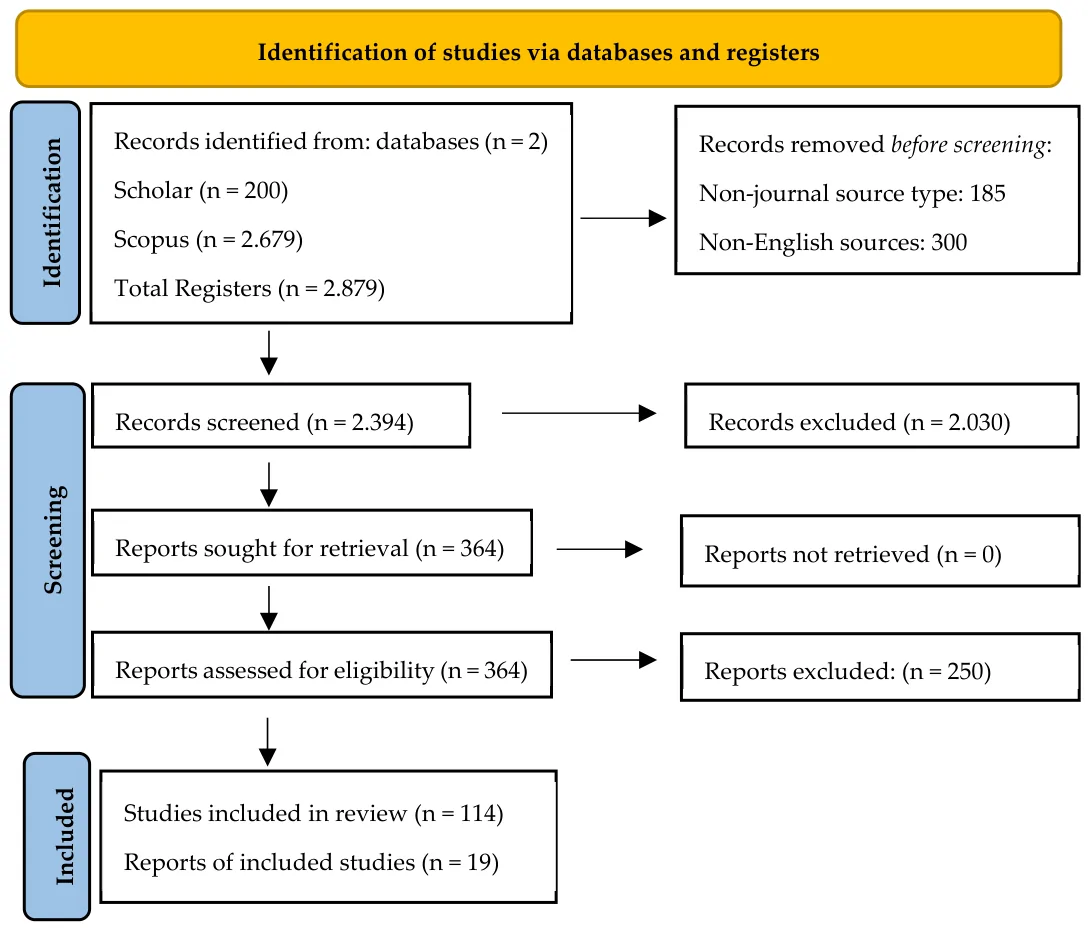
PRISMA flow diagram showing the identification, screening, eligibility, and inclusion of studies [12].

## Data Availability

No new data were created or analyzed in this study. Data sharing is not applicable to this article.

## Abbreviations

The following abbreviations are used in this manuscript:

FAO	Food and agriculture organization
DW	Dry weight
FW	Fresh weight
DPPH	2,2-diphenyl-1-picrylhydrazyl
ABTS	2,2′-azino-bis (3-ethylbenzothiazoline-6-sulfonic acid
FRAP	Ferric reducing antioxidant power
CUPRAC	Cupric reducing antioxidant capacity
UV	Ultraviolet
LC	Liquid chromatography
ESI	Electrospray ionization
LDL	Low-density lipoprotein
